# Differential knockdown of TGF-β ligands in a three-dimensional co-culture tumor- stromal interaction model of lung cancer

**DOI:** 10.1186/1471-2407-14-580

**Published:** 2014-08-09

**Authors:** Masafumi Horie, Akira Saito, Satoshi Noguchi, Yoko Yamaguchi, Mitsuhiro Ohshima, Yasuyuki Morishita, Hiroshi I Suzuki, Tadashi Kohyama, Takahide Nagase

**Affiliations:** Department of Respiratory Medicine, Graduate School of Medicine, The University of Tokyo, 7-3-1 Hongo, Bunkyo-ku, Tokyo, 113-0033 Japan; Division for Health Service Promotion, The University of Tokyo, 7-3-1 Hongo, Bunkyo-ku, Tokyo, 113-0033 Japan; Department of Biochemistry, Nihon University School of Dentistry, 1-8-13 Kanda-Surugadai, Chiyoda-ku, Tokyo, 101-8310 Japan; Department of Biochemistry, Ohu University School of Pharmaceutical Sciences, Misumido 31-1, Tomitamachi, Koriyama, Fukushima, 963-8611 Japan; Department of Molecular Pathology, Graduate School of Medicine, The University of Tokyo, 7-3-1 Hongo, Bunkyo-ku, Tokyo, 113-0033 Japan; The Fourth Department of Internal Medicine, Teikyo University School of Medicine University Hospital, Mizonokuchi, 3-8-3 Mizonokuchi, Takatsu-ku, Kawasaki, Kanagawa, 213-8507 Japan

**Keywords:** RNA interference, MicroRNA, Lentivirus vector, TGF-β, Three-dimensional co-culture, Gel contraction assay

## Abstract

**Background:**

Transforming growth factor (TGF)-β plays a pivotal role in cancer progression through regulating cancer cell proliferation, invasion, and remodeling of the tumor microenvironment. Cancer-associated fibroblasts (CAFs) are the predominant type of stromal cell, in which TGF-β signaling is activated. Among the strategies for TGF-β signaling inhibition, RNA interference (RNAi) targeting of TGF-β ligands is emerging as a promising tool. Although preclinical studies support the efficacy of this therapeutic strategy, its effect on the tumor microenvironment *in vivo* remains unknown. In addition, differential effects due to knockdown of various TGF-β ligand isoforms have not been examined. Therefore, an experimental model that recapitulates tumor–stromal interaction is required for validation of therapeutic agents.

**Methods:**

We have previously established a three-dimensional co-culture model of lung cancer, and demonstrated the functional role of co-cultured fibroblasts in enhancing cancer cell invasion and differentiation. Here, we employed this model to examine how knockdown of TGF-β ligands affects the behavior of different cell types. We developed lentivirus vectors carrying artificial microRNAs against human TGF-β1 and TGF-β2, and tested their effects in lung cancer cells and fibroblasts.

**Results:**

Lentiviral vectors potently and selectively suppressed the expression of TGF-β ligands, and showed anti-proliferative effects on these cells. Furthermore, knockdown of TGF-β ligands attenuated fibroblast-mediated collagen gel contraction, and diminished lung cancer cell invasion in three-dimensional co-culture. We also observed differential effects by targeting different TGF-β isoforms in lung cancer cells and fibroblasts.

**Conclusions:**

Our findings support the notion that RNAi-mediated targeting of TGF-β ligands may be beneficial for lung cancer treatment via its action on both cancer and stromal cells. This study further demonstrates the usefulness of this three-dimensional co-culture model to examine the effect of therapeutic agents on tumor–stromal interaction.

**Electronic supplementary material:**

The online version of this article (doi:10.1186/1471-2407-14-580) contains supplementary material, which is available to authorized users.

## Background

Lung cancer causes the deaths of more than one million people worldwide every year [1]. Despite recent progress in molecular-targeted therapeutics, such as inhibitors of epidermal growth factor receptor (EGFR) tyrosine kinase and anaplastic lymphoma kinase (ALK), failure to achieve long-lasting therapeutic responses has emphasized the need for novel treatment strategies [2, 3].

Most forms of cancer are associated with a stromal response and extracellular matrix (ECM) deposition, referred to as desmoplasia, which is critically regulated by cancer-associated fibroblasts (CAFs) [4]. Cancer tissue remodeling allows tumor cells to grow and disseminate, and contributes to increased interstitial fluid pressure, which can be an obstacle to drug delivery [5].

Among the soluble factors involved in the tumor–stromal interaction, transforming growth factor (TGF)-β plays a pivotal role. In premalignant stages, TGF-β acts as a tumor suppressor by inhibiting proliferation and apoptotic induction in epithelial cells. In later stages, epithelial cells become refractory to the growth inhibitory effect of TGF-β and begin to secrete high levels of TGF-β, which in turn exhibits tumor-promoting activity, such as angiogenesis, immune evasion, fibroblast activation, and ECM accumulation [6–8]. Furthermore, TGF-β increases the migratory and invasive capacity of cancer cells by inducing the epithelial–mesenchymal transition (EMT) [9, 10]. Indeed, TGF-β levels in both serum and tissues were elevated and associated with worsening prognosis in patients with lung cancer [11, 12]. As such, TGF-β may be a promising target for cancer therapy. However, in contrast to cancer cells, the role of TGF-β signaling in the tumor stroma is poorly understood, at least partly due to technical limitations in detecting TGF-β signaling activation *in situ*.

RNA interference (RNAi) has been used widely to induce the potent and specific inhibition of gene expression. Several variants of small regulatory RNAs are involved in RNAi, including synthetic double-stranded small interfering RNAs (siRNAs), RNA polymerase III (pol III)-transcribed small hairpin RNAs (shRNAs), and endogenous or artificial microRNAs (miRNAs) that are transcribed by RNA polymerase II (pol II) as pri-miRNA, and subsequently processed into mature miRNAs [13, 14]. Vectors that enable the expression of engineered miRNA sequences from Pol II promoters have been developed [15], in which the stem sequences of an endogenous miRNA precursor are substituted with unrelated base-paired sequences that target specific genes.

Among the therapeutic strategies for TGF-β signaling inhibition, RNAi is emerging as a promising tool [13]. Recent advances in RNAi technology are overcoming previous obstacles, such as instability *in vivo*, impeded drug delivery, and undesirable off-target effects. In animal experiments, RNAi agents directed against TGF-β ligands have successfully ameliorated outcomes in disease models [16], and raised hope that this approach may be useful in a clinical setting.

However, the three isoforms of TGF-β ligands—TGF-β1, TGF-β2, and TGF-β3—show different expression profiles in various tissues and cell types. To develop effective therapeutic strategies for silencing TGF-β ligands, identifying the appropriate isoform and target cell type may be critical. To our knowledge, the differential effects of eliminating specific TGF-β isoforms in a given tissue type remain unstudied.

In the present study, we explored the therapeutic effect of TGF-β signaling blockade in lung cancer. We previously developed a three-dimensional (3D) co-culture model for evaluation of tumor–stromal interactions [17]. Using this model, we tested the differential effects of silencing TGF-β ligands in A549 lung cancer cells and HFL-1 lung fibroblasts. Among the three isoforms of TGF-β ligands, TGF-β1 and TGF-β2 (but not TGF-β3) are dominantly expressed in these cells [18–20]. Thus we established lentiviral vectors that transduce artificial miRNAs against human TGF-β1 and TGF-β2 as a tool for testing the effects of TGF-β ligand knockdown.

## Methods

### Cell culture

Tissue culture media and supplements were purchased from GIBCO (Life Technologies, Grand Island, NY). A549 human lung adenocarcinoma cells and HFL-1 human lung fibroblasts were purchased from the American Type Culture Collection (Rockville, MD), and were cultured in Dulbecco’s Modified Eagle’s Medium (DMEM) supplemented with 10% fetal bovine serum (FBS). In addition, 293FT cells were obtained from Invitrogen (Carlsbad, CA), and cultured in 100-mm dish coated with collagen type I (IWAKI, Tokyo, Japan) in DMEM with 10% FBS and 1 mM sodium pyruvate.

### Artificial miRNA sequences

The BLOCK-iT™ Pol II miR RNAi Expression Vector Kit with EmGFP (Invitrogen, Carlsbad, CA) was used for RNAi experiments. The design of the expression vector was based on the use of endogenous murine miR-155 flanking sequences. Artificial miRNA sequences targeting human TGF-β ligands were designed using BLOCK-iT™ RNAi Designer (http://rnaidesigner.Invitrogen.com/rnaiexpress/). Four and three pairs of sense and antisense oligonucleotides were designed for targeting human TGF-β1 and β2, respectively (Additional file 1: Table S1).

### Plasmid construction and preparation of viral vectors

The designed oligonucleotides were annealed, followed by ligation into the pcDNA6.2-GW/EmGFP-miR vector (Invitrogen), which facilitates transfer into a suitable destination vector via Gateway recombination reactions. The EmGFP forward sequence primer (5′- GGCATGGACGAGCTGTACAA -3′) was used for sequencing of the miRNA insert fragments, which was performed using an ABI PRISM^®^ 310 Genetic Analyzer. As the control, pcDNA6.2-GW/EmGFP-miR negative control plasmid (Invitrogen) was used. The sequence containing the miRNA coding region was transferred to the lentivirus vector via the Gateway cloning system (Invitrogen). Briefly, the miRNA coding region was subcloned into the entry plasmid pDONR221 (Invitrogen) using Gateway^®^ BP Clonase™ II Enzyme Mix (Invitrogen). The sequences in the entry plasmids were then transferred to the lentiviral expression vector, pCSII-EF-RfA, using Gateway^®^ LR Clonase™ II Enzyme Mix (Invitrogen).

### Lentivirus infection

The recombinant lentivirus was produced by transfection of 293FT cells with the lentiviral expression vectors, pCMV-VSV-G-RSV-Rev, and pCAG-HIVgp, using Lipofectamine 2000 reagent (Invitrogen). After 72 h, the medium was collected, and 1 × 10^5^ of A549 or HFL-1 cells were infected with 500 μL of medium containing lentiviruses. For double knockdown of TGF-β1 and TGF-β2, 250 μL of each lentivirus-containing medium were used. Infection efficiency was assessed by measuring the percentage of EmGFP-positive cells via flow cytometry (EPICS XL System II; Beckman Coulter, Brea, CA), and knockdown efficiency of target gene was analyzed using an enzyme-linked immunosorbent assay (ELISA).

### RT-PCR

Total RNA was extracted using the RNeasy Mini Kit (Qiagen, Tokyo, Japan). The cDNA was synthesized using SuperScript III Reverse Transcriptase (Invitrogen), following the manufacturer’s protocol. Quantitative reverse transcription (RT)-PCR was performed using Mx-3000P (Stratagene, La Jolla, CA) and QuantiTect SYBR Green PCR (Qiagen). Relative mRNA expression was calculated using the *ΔΔC*_*t*_ method, and expression was normalized to that of the glyceraldehyde 3-phosphate dehydrogenase (GAPDH) gene. The specific primers are shown in Additional file 2: Table S2.

### ELISA for TGF-β1 and TGF-β2

A549 and HFL-1 cells were serum-starved for 24 h, and each supernatant was collected. The concentrations of TGF-β1 and TGF-β2 were measured using the Quantikine ELISA for human TGF-β1/TGF-β2 (R&D Systems, Minneapolis, MN), according to the manufacturer’s instructions. Each supernatant was activated by 1 N HCl, followed by neutralization with 1.2 N NaOH/0.5 M HEPES. The optical density of each reaction was measured at 450 nm using a microplate reader (Bio-Rad, Hercules, CA), and corrected against absorption at 570 nm. The data were analyzed using the Microplate Manager III Macintosh data analysis software (Bio-Rad).

### Cell proliferation assay

A549 cells were seeded at a density of 1 × 10^4^/well on 12-well dishes and HFL-1 cells were seeded at 4 × 10^4^/well on 6-well dishes. Both cell types were cultured in DMEM containing 10% FBS. Cells were counted on days 1, 3, and 5 after seeding using a hemocytometer.

### Collagen gel contraction assay and 3D co-culture

Three-dimensional gel cultures were carried out according to the previously published protocol [17]. Briefly, collagen gels were prepared by mixing 0.5 mL of fibroblast cell suspension (~2.5 × 10^5^ cells) in FBS, 2.3 mL of type I collagen (Cell matrix type IA; Nitta Gelatin, Tokyo, Japan), 670 μL of 5× DMEM, and 330 μL of reconstitution buffer, following the manufacturer’s recommendations. The mixture (3 mL) was cast into each well of the six-well culture plates. The solution was then allowed to polymerize at 37°C for 30 min. After overnight incubation, each gel was detached and cultured in growth medium, and the surface area of the gels was quantified via densitometry (Densitograph, ATTO, Tokyo, Japan) for 5 consecutive days, and the final size relative to initial size was determined. For 3D co-culture, A549 cells (2 × 10^5^) were seeded on the surface of each gel prior to overnight incubation. After 5 days of floating culture, the gel was fixed in formalin solution and embedded in paraffin, and vertical sections were stained with hematoxylin and eosin.

### Statistics

Results were confirmed by performing experiments in triplicate. Analyses were performed using JMP version 9 (SAS Institute Inc., Tokyo, Japan). For statistical significance, differences between two experimental groups were examined using Student’s *t*-test, and Dunnett’s test was used for multiple comparisons with control group. *P <* 0.05 was considered to indicate significance.

### GSEA (gene set enrichment analysis)

Navab *et al.* reported gene expression profiles for 15 pairs of lung CAFs and NFs, and identified genes enriched in lung CAFs [21]. GSEA was performed using these microarray data sets (GSE22862) deposited in the public database. To obtain a gene set regulated by TGF-β, we used publicly available microarray datasets, derived from two lung fibroblast cell lines stimulated by TGF-β: HFL-1 (GSE27597) and IMR-90 (GSE17518) [22, 23]. We extracted the top 800 TGF-β-induced genes from each dataset, as identified through the Significance Analysis of Microarrays (SAM) method. Combining these two gene lists, we isolated 196 commonly induced genes in two lung fibroblast cell lines, which were defined as ‘TGF-β-regulated genes’ (Additional file 3: Table S3).

## Results

### TGF-β signaling is activated in lung CAFs

CAFs are a major constituent of the tumor stroma, and we have previously shown that lung CAFs are more potent in enhancing cancer cell invasion and collagen gel contraction than normal lung fibroblasts (NFs) [17]. Although the role of TGF-β in cancer cells and lung fibroblasts has been investigated extensively, TGF-β function in CAFs remains largely unknown due to technical hurdles in isolating fibroblasts from lung cancer tissues.

**Figure 1:**
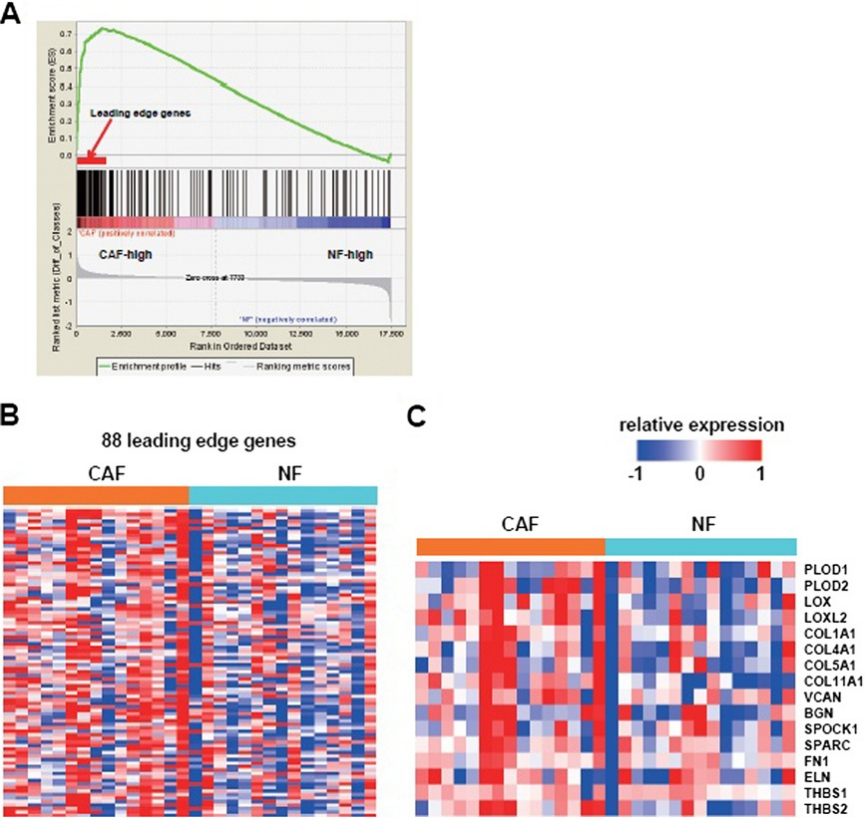
**Gene set enrichment analysis (GSEA). A**: GSEA was used to examine the enrichment of identified TGF-β-regulated genes in CAFs. ‘TGF-β-regulated genes’ include 196 genes induced by TGF-β in both IMR-90 and HFL-1 lung fibroblast cell lines. CAF and NF gene expression profiles reported by Navab *et al.*[21] were used. Enrichment of TGF-β-regulated genes is shown schematically with those that best correlated with the CAF phenotype on the left (‘CAF-high’) and the genes that best correlated with the NF phenotype on the right (‘NF-high’). **B**: A heat map representing the relative expression change of ‘ 88 leading edge genes’ which were obtained by GSEA analysis in CAFs and NFs. **C**: A heat map representing the relative expression change of selected ‘16 ECM related genes’.

To examine TGF-β signaling activation status in lung CAFs, we used gene set enrichment analysis (GSEA) to determine whether the expression of the identified TGF-β-regulated genes was enhanced in lung CAFs compared to NFs. This was performed using microarray data sets of CAFs and NFs reported by Navab *et al*. [21]. These analyses demonstrated that the TGF-β-regulated genes identified through our analysis are in fact highly enriched in CAFs, suggesting that TGF-β signaling is activated in lung CAFs (Figure 1A). We further extracted 88 ‘leading edge genes’ out of the TGF-β-regulated genes. A heatmap of these leading edge genes clearly illustrated differential expression between CAFs and NFs (Figure 1B). As expected, ECM-related genes were enriched among the leading edge genes, and a heatmap of 16 selected ECM related genes apparently showed that TGF-β-regulated ECM-related enzymes and substrates, including PLOD1, LOX, COL1A1, VCAN, SPARC, FN1, ELN, and THBS1, are more enriched in CAFs than NFs (Figure 1C).

### Lentivirus-mediated transduction of artificial miRNAs against human TGF-β1 and TGF-β2

Based on the observation that endogenous TGF-β signaling is activated in lung CAFs, we examined whether TGF-β signaling activation in fibroblasts modulates the behavior of adjacent cancer cells. We also aimed to elucidate the cell-autonomous action of TGF-β in lung cancer cells. To this end, we generated lentiviral vectors that transduced artificial miRNAs against TGF-β ligands, and tested their effects on lung cancer cells and HFL-1 lung fibroblasts. The expression levels of TGF-β isoforms are variable among lung cancer cell lines. In order to survey these differences, we used Cancer Cell Line Encyclopedia (CCLE) data and found that expression of TGF-β isoforms are relatively high in A549 cells among 111 non-small cell lung cancer cell lines (Additional file 4: Figure S1). Therefore, we used A549 lung cancer cells in the following experiments.

Four miRNA sequences were designed to target human TGF-β1, as well as three sequences against TGF-β2 (Additional file 1: Table S1). Next, we determined the efficiency of lentiviral infection by measuring the percentage of EmGFP-positive cells using flow cytometry. More than 95% of A549 cells were positive for EmGFP, suggesting a high transduction efficiency for this miRNA sequence (Additional file 5: Figure S2A, left); we observed similar efficiencies for all miRNA sequences used in this study (Additional file 5: Figure S2A, right). Meanwhile, HFL-1 cells showed more modest (but still sufficient) efficiencies for lentiviral infection (Additional file 5: Figure S2B, left). The percentage of EmGFP-positive cells ranged from 65–85% among the miRNA sequences (Additional file 5: Figure S2B, right).

For double knockdown of TGF-β1 and TGF-β2, two combinations of lentiviruses encoding miRNAs against TGF-β1 and TGF-β2 were co-infected: #2 miRNA against TGF-β1 and #2 miRNA against TGF-β2 (TGF-β1KD #2+ TGF-β2KD #2), or #4 miRNA against TGF-β1 and #3 miRNA against TGF-β2 (TGF-β1KD #4+ TGF-β2KD #3). Co-infection with two different lentiviruses showed similar transduction efficiencies compared to single infections, as determined via EmGFP fluorescence (Additional file 5: Figure S2A, right and Additional file 5: Figure S2B, right).

### Potent and selective knockdown of TGF-β1 and TGF-β2

**Figure 2:**
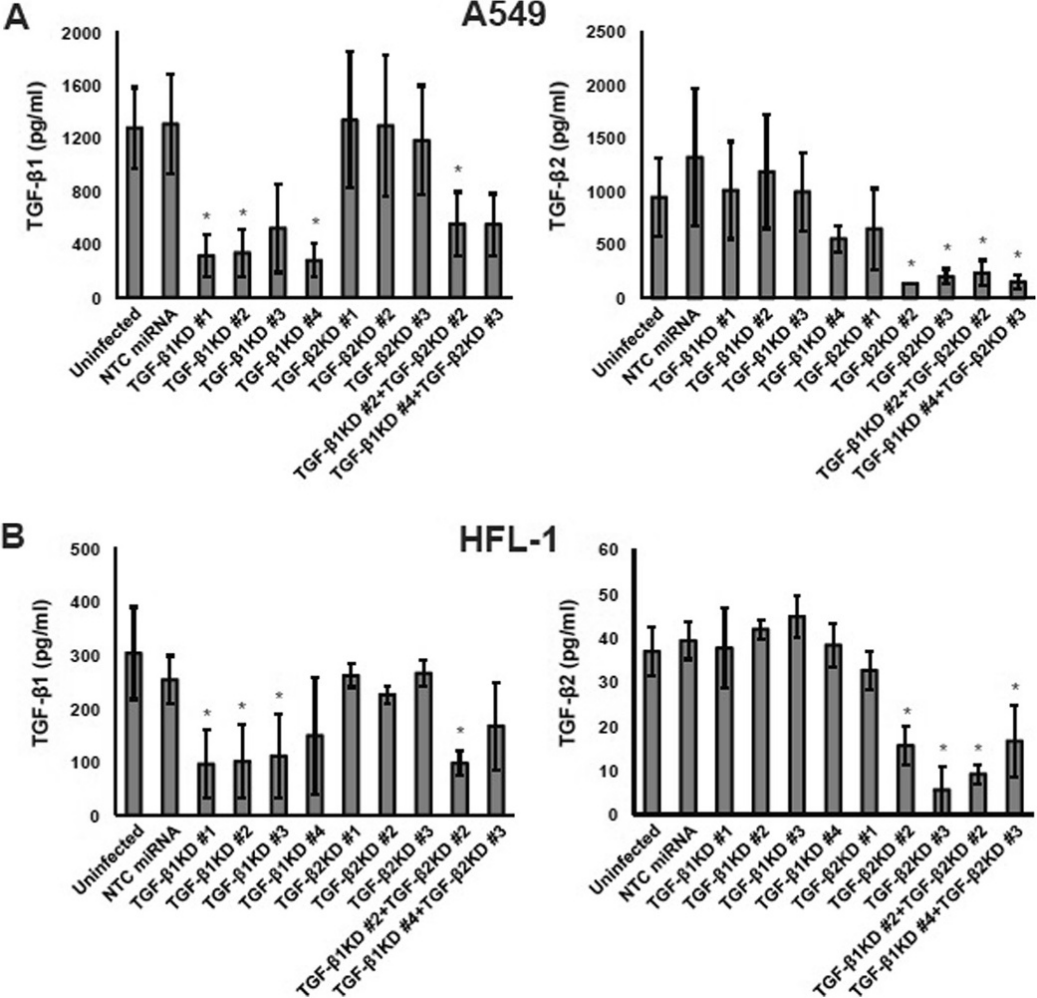
**Knockdown of TGF-β ligands. A**: TGF-β1 and TGF-β2 concentrations measured by ELISA in the supernatant of A549 cells transduced with each miRNA. Left: TGF-β1. Right: TGF-β2. Data shown are the means ± SEM of triplicate analyses. KD: knockdown. NTC: negative control. The concentration of TGF-β1 or TGF-β2 in the supernatant of cells with TGF-β1 and/or TGF-β2 knockdown was compared to that of cells transduced with NTC miRNA. Statistical significance was determined by Dunnett’s test. ^*^ *P <* 0.05. **B**: TGF-β1 and TGF-β2 concentrations measured by ELISA in the supernatant of HFL-1 cells.

Next, we evaluated the efficiency of TGF-β knockdown through measurement of protein expression via ELISA. To control for unintended effects of experimental manipulation, we examined the expression of TGF-β1 and TGF-β2 in uninfected A549 and HFL-1 cells compared to cells infected with negative control (NTC) miRNAs (Figure 2). No significant difference in TGF-β1 or TGF-β2 expression was observed.

In A549 cells, three of four miRNAs against TGF-β1 (#1, #2, and #4) were able to silence TGF-β1 expression, whereas all three miRNAs against TGF-β2 were ineffective for TGF-β1 (Figure 2A, left). Two out of three miRNAs against TGF-β2 (#2 and #3) silenced TGF-β2 expression, whereas all four miRNAs against TGF-β1 were ineffective for TGF-β2 (Figure 2A, right). In HFL-1 cells, three of four miRNAs against TGF-β1 (#1, #2 and #3) were able to silence TGF-β1 expression, whereas all three miRNAs against TGF-β2 were ineffective for TGF-β1 (Figure 2B, left). Two of three miRNAs against TGF-β2 (#2 and #3) silenced TGF-β2 expression, whereas all four miRNAs against TGF-β1 were ineffective for TGF-β2 (Figure 2B, right). These results show that miRNAs against TGF-β1 or TGF-β2 exert their effects in a selective manner for each ligand. Out of the two combinations tested for double knockdown, miRNA #2 against TGF-β1 and #2 against TGF-β2 showed efficient silencing in both A549 and HFL-1 cells (Figure 2). Therefore, we selected miRNA sequences #2 against TGF-β1 and #2 against TGF-β2, for single or double knockdown in the following experiments.

### Cell proliferation is suppressed by knockdown of TGF-β1 and/or TGF-β2

**Figure 3:**
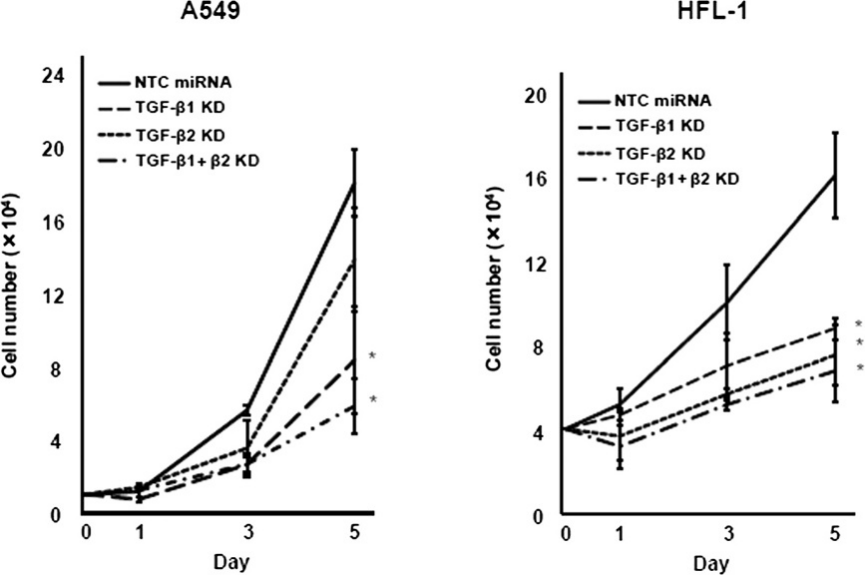
**Cell proliferation assay.** Cell proliferation curve in A549 or HFL-1 cells transduced with NTC miRNA (solid line) compared to cells transduced with miRNA against TGF-β1 (dashed line: TGF-β1 KD), TGF-β2 (dotted line: TGF-β2 KD), or TGF-β1 and TGF-β2 (dashed-dotted line: TGF-β1 + β2 KD). Cell counts were carried out on days 1, 3, and 5 after seeding. Left: A549. Right: HFL-1. Data shown are the means ± SEM of triplicate analyses. Numbers of cells with TGF-β1 and/or TGF-β2 knockdown on day 5 was compared to that in the cells transduced with NTC miRNA. Statistical significance was determined by Student’s *t*-test. ^*^ *P <* 0.05.

Next, we investigated whether TGF-β1 and/or TGF-β2 knockdown affected the proliferation of A549 and HFL-1 cells. In both cell types, the transduction of artificial miRNAs against TGF-β1 or TGF-β2 suppressed cell proliferation (Figure 3), and this anti-proliferative effect was enhanced in cells subject to double knockdown, compared to single knockdown of either TGF-β1 or TGF-β2.

TGF-β is a strong inhibitor of proliferation in most epithelial cells, whereas it promotes proliferation in mesenchymal cells and enhances cancer cell survival [6–8]. Our lentivirus-mediated miRNA delivery system maintains stable knockdown of TGF-β1 and/or TGF-β2. This may alter cell signaling in the steady state and modulate the cell machinery that regulates cell survival or proliferation, thereby resulting in suppressed cell proliferation.

### Altered EMT-related gene expression via TGF-β1 and/or TGF-β2 knockdown

EMT is crucial for cancer cells to acquire invasive phenotypes, which are characterized by downregulation of E-cadherin and upregulation of vimentin. A549 cells stay in an intermediary state of EMT, whereas exogenous TGF-β further promotes acquisition of mesenchymal phenotypes [20]. We examined whether knockdown of TGF-β ligands modulated the expression of EMT markers.

**Figure 4:**
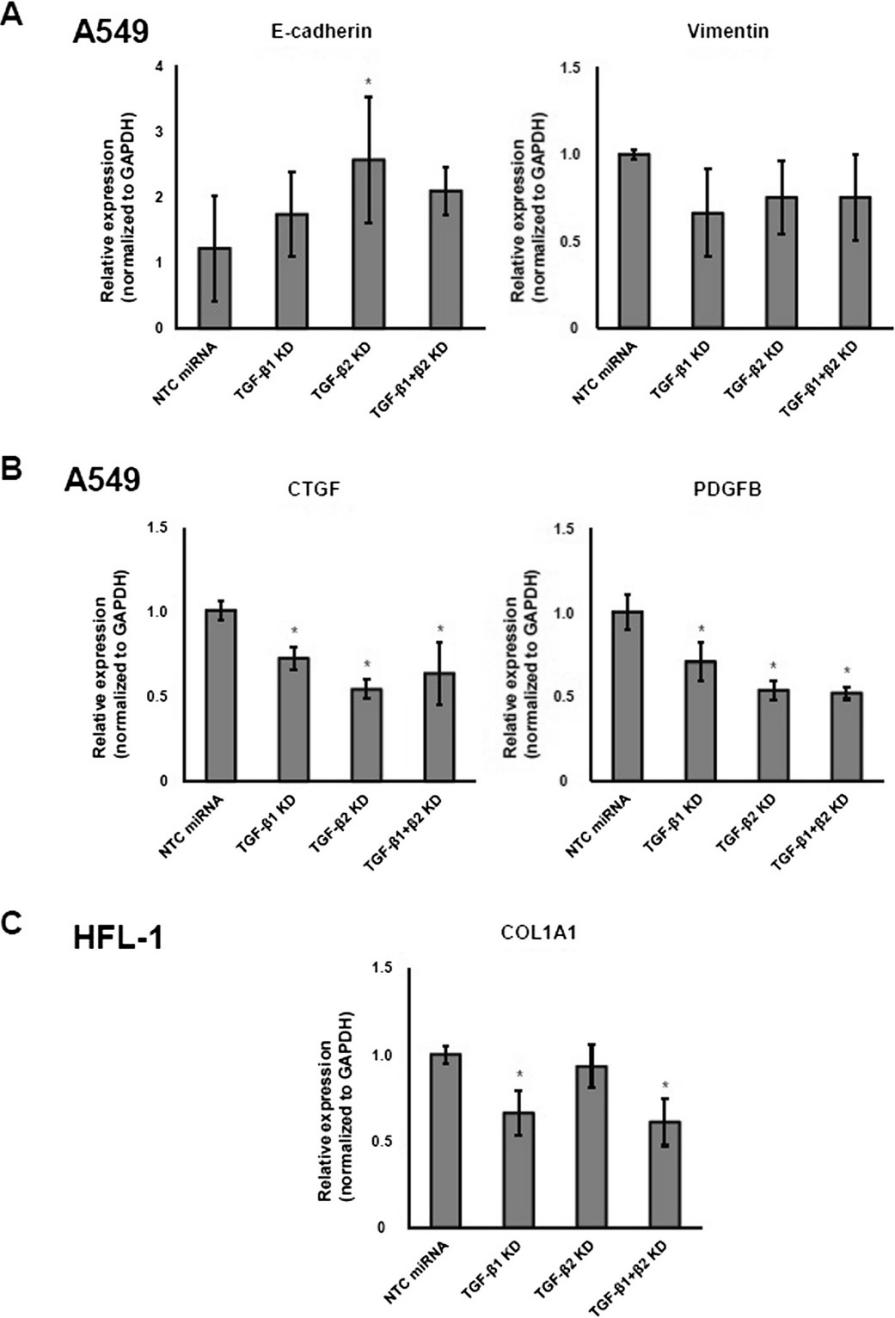
**Quantitative RT-PCR. A**: Quantitative RT-PCR for E-cadherin (left) and vimentin (right) in A549 cells. **B**: Quantitative RT-PCR for CTGF (left) and PDGFB (right) in A549 cells. **C**: Quantitative RT-PCR for COL1A1 in HFL-1 cells. Data shown are the means ± SEM. The relative expression of each gene in cells with TGF-β1 and/or TGF-β2 knockdown was compared to that in the cells transduced with NTC miRNA. Statistical significance was determined by Student’s *t*-test. ^*^ *P <* 0.05.

Silencing of TGF-β2 led to E-cadherin upregulation, suggesting the restoration of epithelial phenotypes. In accordance, vimentin expression was suppressed by knockdown of TGF-β1 and/or TGF-β2, though it failed to reach statistical significance (Figure 4A). These results support the notion that endogenous TGF-β signaling participates in the maintenance of a mesenchymal phenotype in A549 cells in the steady state.

EMT is accompanied by the enhanced expression of fibrogenic growth factors, such as platelet-derived growth factor (PDGF) and connective tissue growth factor (CTGF) [20]. PDGF is a dimeric protein composed of A and B subunits, and it has been reported that the transcription of PDGFB is regulated by TGF-β. Consistent with the previous experiment [20], TGF-β2 silencing led to CTGF downregulation, whereas knockdown of TGF-β1 and/or TGF-β2 attenuated PDGFB expression (Figure 4B).

Upon TGF-β stimulation, fibroblasts convert to an activated phenotype to enhance ECM production. Thus, we examined whether knockdown of TGF-β1 and/or TGF-β2 modulated the expression of α1 (I) collagen (COL1A1), a major component of ECM. In HFL-1 cells, TGF-β1 knockdown decreased the expression of COL1A1, whereas TGF-β2 silencing had no effect (Figure 4C).

These results suggest the differential regulation of target genes by TGF-β1 or TGF-β2 in cancer cells and fibroblasts. During lung branching morphogenesis, TGF-β1 expression is prominent throughout the mesenchyme, whereas TGF-β2 is localized to mainly the epithelium of the developing distal airways [24]. Thus, TGF-β2 may be critical for determining epithelial or cancer cell behavior in a cell-autonomous fashion, whereas endogenous TGF-β1 may play a greater role in fibroblasts.

### TGF-β1 and/or TGF-β2 knockdown attenuates collagen gel contraction in HFL-1 cells

Cancer tissue contraction facilitates tumor progression and contributes to increased interstitial fluid pressure, which hampers drug delivery [5]. The collagen gel contraction assay is used widely to recreate tissue contraction in an experimental setting, and it has been shown that TGF-β stimulates fibroblast-mediated collagen gel contraction [25]. We used this assay to investigate whether knockdown of TGF-β1 and/or TGF-β2 modulated tissue contraction through effects on fibroblasts.

**Figure 5:**
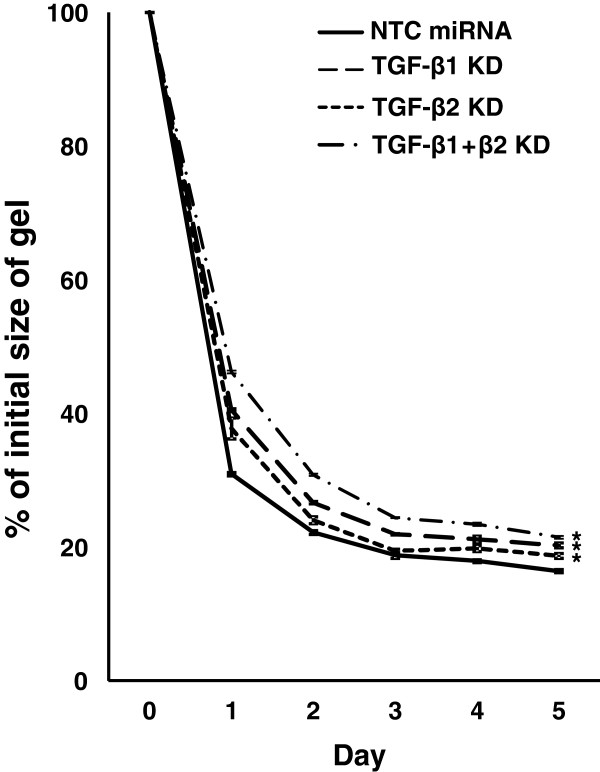
**Collagen gel contraction assay.** Time-course of gel contraction in the presence of HFL-1 transduced with NTC miRNA (solid line), or miRNAs against TGF-β1 (dashed line: TGF-β1 KD), TGF-β2 (dotted line: TGF-β2 KD), or TGF-β1 and TGF-β2 (dashed-dotted line: TGF-β1 + β2 KD). The area of each gel was assessed daily for 5 days and the relative value compared to the initial size was determined. Data shown are the means ± SD of triplicate analyses. Statistical significance was determined by Student’s t-test. *P < 0.05.

Collagen gels were embedded with HFL-1 cells after TGF-β1 and/or TGF-β2 knockdown, and gel size was measured daily. On the first day, the control gel size was reduced to ~50% of the initial value, followed by gradual shrinkage to less than 20% on the fifth day (Figure 5). Compared to the control, knockdown of TGF-β1 and/or TGF-β2 in HFL-1 cells attenuated gel contraction (Figure 5 and Additional file 6: Figure S3). These results suggested that the inhibition of endogenous TGF-β signaling in fibroblasts ameliorates tissue contraction.

### Three-dimensional co-culture of A549 and HFL-1 cells

**Figure 6:**
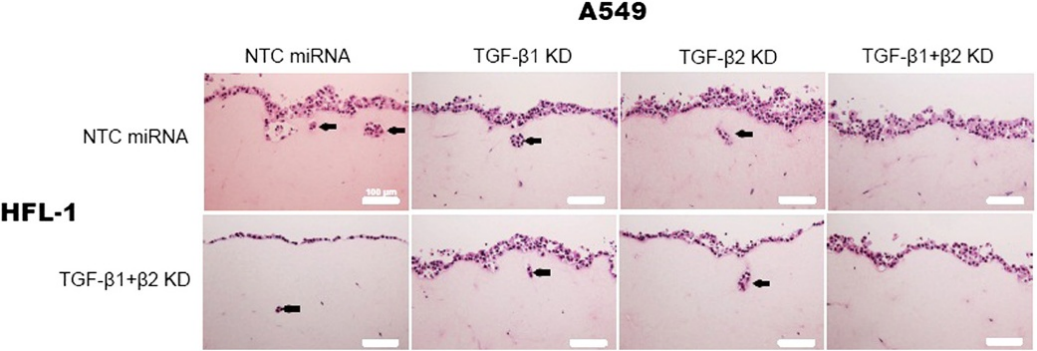
**3D co-culture model.** Hematoxylin and eosin staining of 3D cultured gels composed of A549 and HFL-1 cells transduced with the indicated miRNAs. Upper panels: HFL-1 cells transduced with NTC miRNA. Lower panels: HFL-1 cells transduced with miRNAs against TGF-β1 and TGF-β2 (TGF-β1 + β2 KD). Invading cells are indicated with arrows. Scale bar: 100 μm.

To examine the interaction between lung cancer cells and fibroblasts, we previously established a 3D co-culture model [17]. HFL-1 cells transduced with control miRNAs or those for TGF-β1 and TGF-β2 silencing (double knockdown) were embedded into the collagen gels, and then A549 cells were seeded onto the surface of these gels. The co-cultured collagen gels were subjected to floating culture for an additional 5 days, followed by hematoxylin and eosin staining (Figure 6).

Double knockdown of TGF-β1 and TGF-β2 in HFL-1 cells did not show clear effects on A549 cell invasion, suggesting a minor role for TGF-β produced in HFL-1 cells in this co-culture model (lower panels). In our previous work, we did not examine whether HFL-1 cells enhance lung cancer cell invasion [17], and this study suggests that endogenous TGF-β expression in HFL-1 cells may not have a significant role in invasion promotion.

In contrast, A549 cell invasion was observed when control A549 cells were cultured with control HFL-1 cells (upper left panel). Silencing of either TGF-β1 or TGF-β2 in A549 cells failed to inhibit invasion (upper middle panels), whereas double knockdown of TGF-β1 and TGF-β2 led to complete disappearance of invading cells (upper right panel).

## Discussion

TGF-β plays several crucial roles in cancer progression, affecting both tumor and stromal cells, including fibroblasts [4]. However, very little is known regarding the effects of TGF-β ligand silencing in the context of tumor–stromal or epithelial–mesenchymal interactions [26]. Numerous reports have shown the effects of exogenous TGF-β stimulation in various cell types, whereas the effects of endogenous or cell-autonomous TGF-β signaling are poorly understood. To our knowledge, this study is the first to generate lentiviral vectors encoding artificial miRNAs targeting human TGF-β1 and TGF-β2, and to explore their effects in a co-culture model.

Lentiviral vectors showed efficient transduction in A549 lung cancer cells, as well as HFL-1 lung fibroblasts. Knockdown efficiency to less than 30% of the control was obtained for both TGF-β1 and TGF-β2 in a selective manner. Knockdown of TGF-β ligands suppressed cell proliferation in both A549 and HFL-1 cells. Furthermore, expression of EMT markers and fibrogenic growth factors was modulated in A549 cells, whereas collagen I was downregulated in HFL-1 cells. With regard to cellular function, silencing of TGF-β ligands attenuated HFL-1-mediated collagen gel contraction, and inhibited A549 cell invasion in the 3D co-culture model. All of these findings support the tumor-promoting role of TGF-β, and that the reported beneficial effects of TGF-β inhibition in cancer therapeutics may derive from interfering with tumor–stromal communications.

In our experiments, it appeared that both TGF-β1 and TGF-β2 were abundantly produced in A549 cells, whereas the concentration of TGF-β1 was higher than that of TGF-β2 in the supernatant of HFL-1 cells. Compared to single knockdown, double knockdown of TGF-β1 and TGF-β2 showed stronger effects in A549 cell proliferation and invasion in a 3D co-culture. In HFL-1 cells, TGF-β1 knockdown was more effective than TGF-β2 knockdown in suppressing COL1A1 expression.

Little is known regarding the expression profiles of TGF-β isoforms in various lung cancer cell types. As shown here, knockdown of each TGF-β ligand modulated phenotype in a cell-type-dependent manner. These effects may be much more complicated and variable depending on the multicellular context; nevertheless, our results demonstrate the important role for TGF-β signaling in the tumor microenvironment.

We have reported previously that lung CAFs enhance cancer cell invasion [17]. In the present study, double knockdown of TGF-β1 and TGF-β2 in HFL-1 cells did not show clear effects on A549 cell invasion, and endogenous TGF-β expression in HFL-1 cells seemed to have little effect on lung cancer cell invasion. The precise mechanism underlying CAF-enhanced lung cancer cell invasion remains to be elucidated, and further studies are necessary to clarify the mechanisms underlying cell invasion in our experimental model.

There have been several attempts to exploit TGF-β signaling inhibition as a therapeutic approach for malignant tumors, including the use of TGF-β receptor kinase inhibitors, TGF-β neutralizing antibodies, TGF-β antisense oligonucleotides (AONs), and siRNAs [27]. TGF-β type I receptor kinase inhibitor has been tested for non-small cell lung cancer (NSCLC) patients in a phase II study, but failed to yield clinical benefits [28]. Several animal models of cancer have demonstrated the therapeutic effect of TGF-β neutralizing antibodies [29].

Recently, AONs against TGF-β ligands have shown promising clinical results. Trabedersen (AP 12009) is an AON against human TGF-β2. Intra-tumoral administration of trabedersen in patients with high-grade gliomas led to better tumor control and prolonged survival with fewer adverse events, which prompted a larger phase III trial [30]. Intravenous application of trabedersen in patients with other cancer types is also under evaluation. AP 11014, another AON targeting human TGF-β1, is currently in preclinical development for NSCLC treatment. Furthermore, a phase II trial for belagenpumatucel-L, a vaccine produced from NSCLC cells transfected with TGF-β2 AON, has shown beneficial effects on survival without any significant adverse effects; phase III studies in lung cancer patients are ongoing [31]. RNAi targeting TGF-β ligands is also emerging as a promising tool [13]. In animal experiments, RNAi agents against TGF-β1 demonstrated therapeutically beneficial effects, supporting progression toward future clinical applications [16].

This body of work demonstrates the intensifying interest in TGF-β ligand silencing as a therapeutic approach for lung cancer. To validate therapeutic strategies against TGF-β ligands, it may be critical to target the appropriate TGF-β isoform in a given cell type. The present study provides a useful experimental model to investigate the effect of therapeutic agents targeting TGF-β ligands. Our results suggest that targeting both TGF-β1 and TGF-β2 in lung cancer cells is more effective than single knockdown. Furthermore, TGF-β2 knockdown may play a more specific role in lung cancer cells than in stromal cells, such as fibroblasts. Future studies are warranted to further elucidate the therapeutical benefits of strategies against the different TGF-β ligands.

## Conclusion

Because TGF-β exerts it pleiotropic effects in a variety of cells in the tumor microenvironment, it is useful to evaluate the action of anti-TGF-β therapeutic agents in multicellular culture conditions. Our 3D co-culture model, demonstrated here, represents a useful tool for evaluating differential effects on cancer cells and fibroblasts. In summary, we established a lentivirus-mediated knockdown system for TGF-β ligands, which revealed their multifaceted effects on cell proliferation, EMT, invasion, and ECM remodeling.

## Electronic supplementary material

Additional file 1: Table S1: Sequences of artificial miRNAs against TGF-β ligands. (DOC 36 KB)

Additional file 2: Table S2: Primers for RT-PCR. (DOC 29 KB)

Additional file 3: Table S3: The 196 ‘TGF-β-regulated genes’. (DOC 176 KB)

Additional file 4: Figure S1: Expression levels of TGF-β isoforms in non-small cell lung cancer cell lines. The transcription levels of TGF-β1 and TGF-β2 in non-small cell lung cancer cell lines were retrieved from Cancer Cell Line Encyclopedia (CCLE) database and shown in a scatter plot. A549 cells showed relatively higher levels of TGF-β1 and TGF-β2. (PDF 143 KB)

Additional file 5: Figure S2: Transduction efficiency of lentiviral vectors. A: Transduction efficiency of miRNAs in A549 cells. Left: miRNA transduction was tracked by detecting EmGFP-positive cells using the FL-1 channel of a flow cytometer. A representative result of #2 miRNA transduction against TGF-β1 is shown. The grey and black peaks are from uninfected and lentivirus-transduced cells, respectively. Right: transduction efficiency of each miRNA. KD: knockdown. NTC: negative control. B: Transduction efficiency of miRNA in HFL-1 cells. (PDF 250 KB)

Additional file 6: Figure S3: Collagen gel contraction assay. Photographs of the gels on day 5 in the experiments shown in Figure 5. Identically sized white circles in each well are shown to demonstrate the differences in gel size. (PDF 308 KB)

## Abbreviations

TGF-β: Transforming growth factor-β
EGFR: Epidermal growth factor receptor
ALK: Anaplastic lymphoma kinase
CAFs: Cancer-associated fibroblasts
EMT: Epithelial-mesenchymal transition
ECM: Extracellular matrix
GSEA: Gene set enrichment analysis
PDGF: Platelet-derived growth factor
CTGF: Connective tissue growth factor
RNAi: RNA interference
AON: Antisense oligonucleotides.
