# Supplementary material for: Differential knockdown of TGF-β ligands in a three-dimensional co-culture tumor- stromal interaction model of lung cancer
Source: BMC Cancer. 2014 Aug 9;14:580. doi: 10.1186/1471-2407-14-580 (PMC4132906; doi:10.1186/1471-2407-14-580)
Supplement: Supplementary file 1 — Additional file 1: Table S1: Sequences of artificial miRNAs against TGF-β ligands. (DOC 36 KB) [file 12885_2014_4753_MOESM1_ESM.doc]

**Supplementary Table 1. Sequences of artificial miRNAs against TGF-β ligands**

|  | Oligonucleotide (5’ to 3’) |
| --- | --- |
| TGF-β1 #1 sense | TGCTGATTAGCACGCGGGTGACCTCCGTTTTGGCCACTGACTGACGGAGGTCACGCGTGCTAAT |
| TGF-β1 #1 antisense | CCTGATTAGCACGCGTGACCTCCGTCAGTCAGTGGCCAAAACGGAGGTCACCCGCGTGCTAATC |
| TGF-β1 #2 sense | TGCTGATTTCTGGTACAGCTCCACGTGTTTTGGCCACTGACTGACACGTGGAGGTACCAGAAAT |
| TGF-β1 #2 antisense | CCTGATTTCTGGTACCTCCACGTGTCAGTCAGTGGCCAAAACACGTGGAGCTGTACCAGAAATC |
| TGF-β1 #3 sense | TGCTGTATCCCTGCTGTCACAGGAGCGTTTTGGCCACTGACTGACGCTCCTGTCAGCAGGGATA |
| TGF-β1 #3 antisense | CCTGTATCCCTGCTGACAGGAGCGTCAGTCAGTGGCCAAAACGCTCCTGTGACAGCAGGGATAC |
| TGF-β1 #4 sense | TGCTGATGAGAAGCAGGAAAGGCCGGGTTTTGGCCACTGACTGACCCGGCCTTCTGCTTCTCAT |
| TGF-β1 #4 antisense | CCTGATGAGAAGCAGAAGGCCGGGTCAGTCAGTGGCCAAAACCCGGCCTTTCCTGCTTCTCATC |
| TGF-β2 #1 sense | TGCTGTATCCATTTCCACCCTAGATCGTTTTGGCCACTGACTGACGATCTAGGGGAAATGGATA |
| TGF-β2 #1 antisense | CCTGTATCCATTTCCCCTAGATCGTCAGTCAGTGGCCAAAACGATCTAGGGTGGAAATGGATAC |
| TGF-β2 #2 sense | TGCTGTTCATGAACAGCATCAGTTACGTTTTGGCCACTGACTGACGTAACTGACTGTTCATGAA |
| TGF-β2 #2 antisense | CCTGTTCATGAACAGTCAGTTACGTCAGTCAGTGGCCAAAACGTAACTGATGCTGTTCATGAAC |
| TGF-β2 #3 sense | TGCTGTTCTGATCACCACTGGTATATGTTTTGGCCACTGACTGACATATACCAGGTGATCAGAA |
| TGF-β2 #3 antisense | CCTGTTCTGATCACCTGGTATATGTCAGTCAGTGGCCAAAACATATACCAGTGGTGATCAGAAC |
