# Supplementary material for: Differential knockdown of TGF-β ligands in a three-dimensional co-culture tumor- stromal interaction model of lung cancer
Source: BMC Cancer. 2014 Aug 9;14:580. doi: 10.1186/1471-2407-14-580 (PMC4132906; doi:10.1186/1471-2407-14-580)
Supplement: Supplementary file 2 — Additional file 2: Table S2: Primers for RT-PCR. (DOC 29 KB) [file 12885_2014_4753_MOESM2_ESM.doc]

Supplementary Table 2. Primers for RT-PCR

| Gene | Forward Primer (5’ to 3’) | Reverse Primer (5’ to 3’) |
| --- | --- | --- |
| GAPDH | GGTGAAGGTCGGAGTCAACGGA | GAGGGATCTCGCTCCTGGAAGA |
| COL1A1 | AGGACAAGAGGCATGTCTGGTT | TTGCAGTGGTAGGTGATGTTCTG |
| PDGFB | GTTTGCTGTTGAGGTGGCTGTAGATG | GAGATGAAAGGAACCAGAGGAAGAG |
| CTGF | CAGCATGGACGTTCGTCTG | AACCACGGTTTGGTCCTTGG |
| CDH1  (E-cadherin) | CCCATCAGCTGCCCAGAAAATGA | CTGTCACCTTCAGCCATCCTGTTT |
| VIM  (Vimentin) | GACAATGCGTCTCTGGCACGTCT | TTCTTCTGCCTCCTGCAGGTTCT |
