# Supplementary material for: Differential knockdown of TGF-β ligands in a three-dimensional co-culture tumor- stromal interaction model of lung cancer
Source: BMC Cancer. 2014 Aug 9;14:580. doi: 10.1186/1471-2407-14-580 (PMC4132906; doi:10.1186/1471-2407-14-580)
Supplement: Supplementary file 3 — Additional file 3: Table S3: The 196 ‘TGF-β-regulated genes’. (DOC 176 KB) [file 12885_2014_4753_MOESM3_ESM.doc]

Supplementary Table 3. The 196 ‘TGF-β-regulated genes’

| Gene symbol | Gene Name | Entrez  Gene |
| --- | --- | --- |
| ACLY | ATP citrate lyase | 47 |
| ACSL3 | acyl-CoA synthetase long-chain family member 3 | 2181 |
| ACTA2 | actin, alpha 2, smooth muscle, aorta | 59 |
| ACTB | actin, beta | 60 |
| ACTG2 | actin, gamma 2, smooth muscle, enteric | 72 |
| ADAM19 | ADAM metallopeptidase domain 19 | 8728 |
| ALDH18A1 | aldehyde dehydrogenase 18 family, member A1 | 5832 |
| ALDH1B1 | aldehyde dehydrogenase 1 family, member B1 | 219 |
| AMIGO2 | adhesion molecule with Ig-like domain 2 | 347902 |
| APBB2 | amyloid beta (A4) precursor protein-binding, family B, member 2 | 323 |
| ARF4 | ADP-ribosylation factor 4 | 378 |
| ASNS | asparagine synthetase (glutamine-hydrolyzing) | 440 |
| ASS1 | argininosuccinate synthase 1 | 445 |
| B4GALT4 | UDP-Gal:betaGlcNAc beta 1,4- galactosyltransferase, polypeptide 4 | 8702 |
| BGN | biglycan | 633 |
| C19orf10 | chromosome 19 open reading frame 10 | 56005 |
| CALD1 | caldesmon 1 | 800 |
| CALU | calumenin | 813 |
| CAP1 | CAP, adenylate cyclase-associated protein 1 (yeast) | 10487 |
| CARS | cysteinyl-tRNA synthetase | 833 |
| CAV2 | caveolin 2 | 858 |
| CBS | cystathionine-beta-synthase | 875 |
| CD55 | CD55 molecule, decay accelerating factor for complement (Cromer blood group) | 1604 |
| CDC42EP3 | CDC42 effector protein (Rho GTPase binding) 3 | 10602 |
| CDKN2B | cyclin-dependent kinase inhibitor 2B (p15, inhibits CDK4) | 1030 |
| CEBPG | CCAAT/enhancer binding protein (C/EBP), gamma | 1054 |
| CHAC1 | ChaC, cation transport regulator homolog 1 (E. coli) | 79094 |
| CLINT1 | clathrin interactor 1 | 9685 |
| CNN1 | calponin 1, basic, smooth muscle | 1264 |
| CNN3 | calponin 3, acidic | 1266 |
| COL11A1 | collagen, type XI, alpha 1 | 1301 |
| COL1A1 | collagen, type I, alpha 1 | 1277 |
| COL4A1 | collagen, type IV, alpha 1 | 1282 |
| COL5A1 | collagen, type V, alpha 1 | 1289 |
| COMP | cartilage oligomeric matrix protein | 1311 |
| CORO1C | coronin, actin binding protein, 1C | 23603 |
| CREB3L1 | cAMP responsive element binding protein 3-like 1 | 90993 |
| CRIM1 | cysteine rich transmembrane BMP regulator 1 (chordin-like) | 51232 |
| CSRP1 | cysteine and glycine-rich protein 1 | 1465 |
| CTPS1 | CTP synthase 1 | 1503 |
| CYR61 | cysteine-rich, angiogenic inducer, 61 | 3491 |
| DDAH1 | dimethylarginine dimethylaminohydrolase 1 | 23576 |
| DDIT4 | DNA-damage-inducible transcript 4 | 54541 |
| DKK1 | dickkopf 1 homolog (Xenopus laevis) | 22943 |
| DKK2 | dickkopf 2 homolog (Xenopus laevis) | 27123 |
| DNAJC10 | DnaJ (Hsp40) homolog, subfamily C, member 10 | 54431 |
| DPYSL3 | dihydropyrimidinase-like 3 | 1809 |
| DYNLT3 | dynein, light chain, Tctex-type 3 | 6990 |
| EDEM1 | ER degradation enhancer, mannosidase alpha-like 1 | 9695 |
| EIF2S2 | eukaryotic translation initiation factor 2, subunit 2 beta, 38kDa | 8894 |
| EIF4EBP1 | eukaryotic translation initiation factor 4E binding protein 1 | 1978 |
| ELN | Elastin | 2006 |
| EMC1 | ER membrane protein complex subunit 1 | 23065 |
| ENTPD7 | ectonucleoside triphosphate diphosphohydrolase 7 | 57089 |
| EPRS | glutamyl-prolyl-tRNA synthetase | 2058 |
| FAM98A | family with sequence similarity 98, member A | 25940 |
| FBN1 | fibrillin 1 | 2200 |
| FERMT2 | fermitin family member 2 | 10979 |
| FKBP14 | FK506 binding protein 14, 22 kDa | 55033 |
| FLNA | filamin A, alpha | 2316 |
| FLNB | filamin B, beta | 2317 |
| FN1 | fibronectin 1 | 2335 |
| FST | Follistatin | 10468 |
| FSTL1 | follistatin-like 1 | 11167 |
| GARS | glycyl-tRNA synthetase | 2617 |
| GBP1 | guanylate binding protein 1, interferon-inducible | 2633 |
| GOLT1B | golgi transport 1B | 51026 |
| GRAMD3 | GRAM domain containing 3 | 65983 |
| HMGCR | 3-hydroxy-3-methylglutaryl-CoA reductase | 3156 |
| HSP90B1 | heat shock protein 90kDa beta (Grp94), member 1 | 7184 |
| HSPA13 | heat shock protein 70kDa family, member 13 | 6782 |
| HSPA5 | heat shock 70kDa protein 5 (glucose-regulated protein, 78kDa) | 3309 |
| IARS | isoleucyl-tRNA synthetase | 3376 |
| IDI1 | isopentenyl-diphosphate delta isomerase 1 | 3422 |
| IGFBP3 | insulin-like growth factor binding protein 3 | 3486 |
| IGFBP7 | insulin-like growth factor binding protein 7 | 3490 |
| INSIG1 | insulin induced gene 1 | 3638 |
| ITCH | itchy E3 ubiquitin protein ligase | 83737 |
| ITGA5 | integrin, alpha 5 (fibronectin receptor, alpha polypeptide) | 3678 |
| ITGA7 | integrin, alpha 7 | 3679 |
| ITGAV | integrin, alpha V | 3685 |
| ITGBL1 | integrin, beta-like 1 (with EGF-like repeat domains) | 9358 |
| KDELC1 | KDEL (Lys-Asp-Glu-Leu) containing 1 | 79070 |
| KRT18 | keratin 18 | 3875 |
| LDHA | lactate dehydrogenase A | 3939 |
| LDLR | low density lipoprotein receptor | 3949 |
| LEPRE1 | leucine proline-enriched proteoglycan (leprecan) 1 | 64175 |
| LEPREL1 | leprecan-like 1 | 55214 |
| LEPREL4 | leprecan-like 4 | 10609 |
| LIMS1 | LIM and senescent cell antigen-like domains 1 | 3987 |
| LIMS2 | LIM and senescent cell antigen-like domains 2 | 55679 |
| LMCD1 | LIM and cysteine-rich domains 1 | 29995 |
| LOX | lysyl oxidase | 4015 |
| LOXL2 | lysyl oxidase-like 2 | 4017 |
| LRRC59 | leucine rich repeat containing 59 | 55379 |
| MAK16 | MAK16 homolog (S. cerevisiae) | 84549 |
| MAP3K7CL | MAP3K7 c-terminal like | 56911 |
| MARS | methionyl-tRNA synthetase | 4141 |
| MCAM | melanoma cell adhesion molecule | 4162 |
| MICAL2 | microtubule associated monoxygenase, calponin and LIM domain containing 2 | 9645 |
| MLLT11 | myeloid/lymphoid or mixed-lineage leukemia (trithorax homolog, Drosophila); translocated to, 11 | 10962 |
| MORC4 | MORC family CW-type zinc finger 4 | 79710 |
| MPZL1 | myelin protein zero-like 1 | 9019 |
| MRC2 | mannose receptor, C type 2 | 9902 |
| MRPL3 | mitochondrial ribosomal protein L3 | 11222 |
| MSMO1 | methylsterol monooxygenase 1 | 6307 |
| MTHFD2 | methylenetetrahydrofolate dehydrogenase (NADP+ dependent) 2, methenyltetrahydrofolate cyclohydrolase | 10797 |
| MTMR2 | myotubularin related protein 2 | 8898 |
| MVD | mevalonate (diphospho) decarboxylase | 4597 |
| MYL9 | myosin, light chain 9, regulatory | 10398 |
| MYO1E | myosin IE | 4643 |
| NANS | N-acetylneuraminic acid synthase | 54187 |
| NAV3 | neuron navigator 3 | 89795 |
| NEDD9 | neural precursor cell expressed, developmentally down-regulated 9 | 4739 |
| NNMT | nicotinamide N-methyltransferase | 4837 |
| NT5DC2 | 5'-nucleotidase domain containing 2 | 64943 |
| ORMDL2 | ORM1-like 2 (S. cerevisiae) | 29095 |
| P4HA1 | prolyl 4-hydroxylase, alpha polypeptide I | 5033 |
| P4HA2 | prolyl 4-hydroxylase, alpha polypeptide II | 8974 |
| P4HB | prolyl 4-hydroxylase, beta polypeptide | 5034 |
| PAICS | phosphoribosylaminoimidazole carboxylase, phosphoribosylaminoimidazole succinocarboxamide synthetase | 10606 |
| PALLD | palladin, cytoskeletal associated protein | 23022 |
| PAWR | PRKC, apoptosis, WT1, regulator | 5074 |
| PCK2 | phosphoenolpyruvate carboxykinase 2 (mitochondrial) | 5106 |
| PDGFC | platelet derived growth factor C | 56034 |
| PDIA4 | protein disulfide isomerase family A, member 4 | 9601 |
| PDIA6 | protein disulfide isomerase family A, member 6 | 10130 |
| PDLIM5 | PDZ and LIM domain 5 | 10611 |
| PELO | pelota homolog (Drosophila) | 53918 |
| PGM3 | phosphoglucomutase 3 | 5238 |
| PHLDA2 | pleckstrin homology-like domain, family A, member 2 | 7262 |
| PLAUR | plasminogen activator, urokinase receptor | 5329 |
| PLOD1 | procollagen-lysine, 2-oxoglutarate 5-dioxygenase 1 | 5351 |
| PLOD2 | procollagen-lysine, 2-oxoglutarate 5-dioxygenase 2 | 5352 |
| PMAIP1 | phorbol-12-myristate-13-acetate-induced protein 1 | 5366 |
| PRPS1 | phosphoribosyl pyrophosphate synthetase 1 | 5631 |
| PRSS23 | protease, serine, 23 | 11098 |
| PRUNE2 | prune homolog 2 (Drosophila) | 158471 |
| PSAT1 | phosphoserine aminotransferase 1 | 29968 |
| PTPLB | protein tyrosine phosphatase-like (proline instead of catalytic arginine), member b | 201562 |
| PTPRF | protein tyrosine phosphatase, receptor type, F | 5792 |
| PTRF | polymerase I and transcript release factor | 284119 |
| PTS | 6-pyruvoyltetrahydropterin synthase | 5805 |
| PXDC1 | PX domain containing 1 | 221749 |
| PYCR1 | pyrroline-5-carboxylate reductase 1 | 5831 |
| RAB3B | RAB3B, member RAS oncogene family | 5865 |
| RAI14 | retinoic acid induced 14 | 26064 |
| RCN3 | reticulocalbin 3, EF-hand calcium binding domain | 57333 |
| RDH11 | retinol dehydrogenase 11 (all-trans/9-cis/11-cis) | 51109 |
| SEC23B | Sec23 homolog B (S. cerevisiae) | 10483 |
| SEL1L3 | sel-1 suppressor of lin-12-like 3 (C. elegans) | 23231 |
| SEMA3C | sema domain, immunoglobulin domain (Ig), short basic domain, secreted, (semaphorin) 3C | 10512 |
| SERPINE1 | serpin peptidase inhibitor, clade E (nexin, plasminogen activator inhibitor type 1), member 1 | 5054 |
| SERPINE2 | serpin peptidase inhibitor, clade E (nexin, plasminogen activator inhibitor type 1), member 2 | 5270 |
| SERPINH1 | serpin peptidase inhibitor, clade H (heat shock protein 47), member 1, (collagen binding protein 1) | 871 |
| SH3PXD2A | SH3 and PX domains 2A | 9644 |
| SHMT2 | serine hydroxymethyltransferase 2 (mitochondrial) | 6472 |
| SLC16A1 | solute carrier family 16, member 1 (monocarboxylic acid transporter 1) | 6566 |
| SLC1A4 | solute carrier family 1 (glutamate/neutral amino acid transporter), member 4 | 6509 |
| SLC31A1 | solute carrier family 31 (copper transporters), member 1 | 1317 |
| SLC38A1 | solute carrier family 38, member 1 | 81539 |
| SLC39A14 | solute carrier family 39 (zinc transporter), member 14 | 23516 |
| SLC7A1 | solute carrier family 7 (cationic amino acid transporter, y+ system), member 1 | 6541 |
| SLC7A5 | solute carrier family 7 (amino acid transporter light chain, L system), member 5 | 8140 |
| SPARC | secreted protein, acidic, cysteine-rich (osteonectin) | 6678 |
| SPOCK1 | sparc/osteonectin, cwcv and kazal-like domains proteoglycan (testican) 1 | 6695 |
| SQLE | squalene epoxidase | 6713 |
| SRPX2 | sushi-repeat containing protein, X-linked 2 | 27286 |
| SSR1 | signal sequence receptor, alpha | 6745 |
| SSR3 | signal sequence receptor, gamma (translocon-associated protein gamma) | 6747 |
| STK17B | serine/threonine kinase 17b | 9262 |
| SULF1 | sulfatase 1 | 23213 |
| SYNE1 | spectrin repeat containing, nuclear envelope 1 | 23345 |
| SYNGR2 | synaptogyrin 2 | 9144 |
| SYT1 | synaptotagmin I | 6857 |
| TAGLN | Transgelin | 6876 |
| TARS | threonyl-tRNA synthetase | 6897 |
| THBS1 | thrombospondin 1 | 7057 |
| THBS2 | thrombospondin 2 | 7058 |
| TM6SF1 | transmembrane 6 superfamily member 1 | 53346 |
| TMEM2 | transmembrane protein 2 | 23670 |
| TMEM45A | transmembrane protein 45A | 55076 |
| TNFRSF12A | tumor necrosis factor receptor superfamily, member 12A | 51330 |
| TNFSF4 | tumor necrosis factor (ligand) superfamily, member 4 | 7292 |
| TPM1 | tropomyosin 1 (alpha) | 7168 |
| TRIB3 | tribbles homolog 3 (Drosophila) | 57761 |
| TSPAN13 | tetraspanin 13 | 27075 |
| TSTA3 | tissue specific transplantation antigen P35B | 7264 |
| TUFT1 | tuftelin 1 | 7286 |
| UCK2 | uridine-cytidine kinase 2 | 7371 |
| UGGT2 | UDP-glucose glycoprotein glucosyltransferase 2 | 55757 |
| VCAN | Versican | 1462 |
| WDR1 | WD repeat domain 1 | 9948 |
| WDR41 | WD repeat domain 41 | 55255 |
| YIF1A | Yip1 interacting factor homolog A (S. cerevisiae) | 10897 |
| YKT6 | YKT6 v-SNARE homolog (S. cerevisiae) | 10652 |
