# Supplementary figures and images for: Differential knockdown of TGF-β ligands in a three-dimensional co-culture tumor- stromal interaction model of lung cancer
Source: BMC Cancer. 2014 Aug 9;14:580. doi: 10.1186/1471-2407-14-580 (PMC4132906; doi:10.1186/1471-2407-14-580)

Supplementary Figure 1

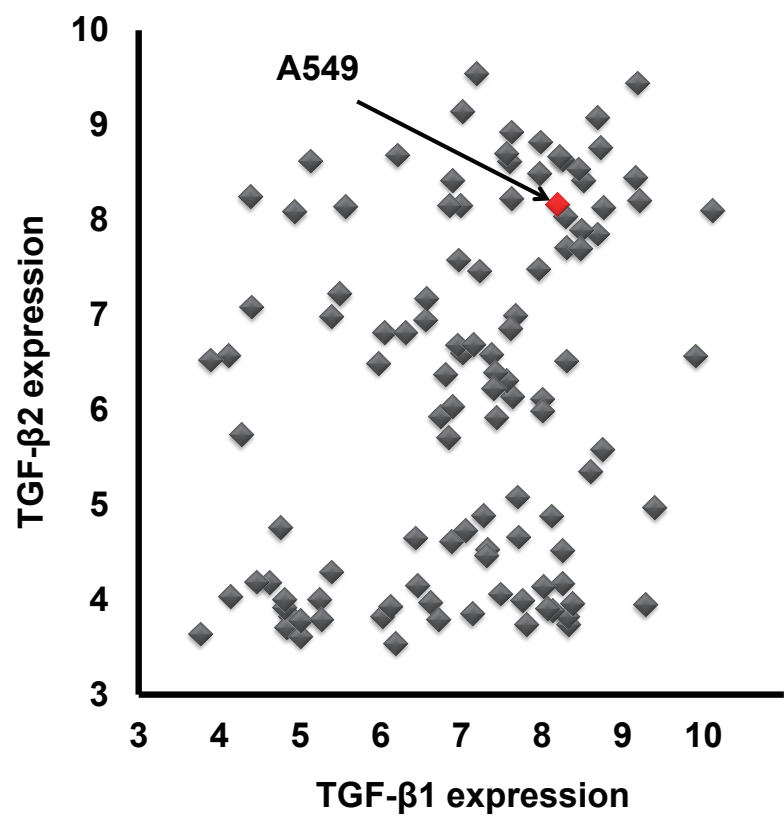

Supplement: Supplementary file 4 — Additional file 4: Figure S1: Expression levels of TGF-β isoforms in non-small cell lung cancer cell lines. The transcription levels of TGF-β1 and TGF-β2 in non-small cell lung cancer cell lines were retrieved from Cancer Cell Line Encyclopedia (CCLE) database and shown in a scatter plot. A549 cells showed relatively higher levels of TGF-β1 and TGF-β2. (PDF 143 KB) [file 12885_2014_4753_MOESM4_ESM.pdf]

Supplementary Figure 2

A A549

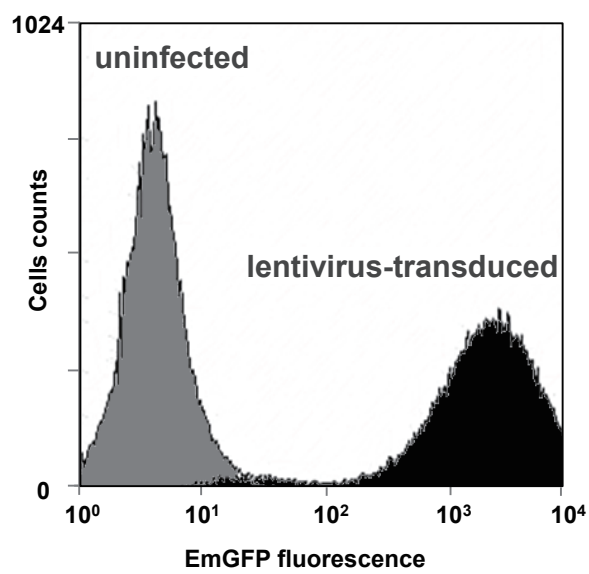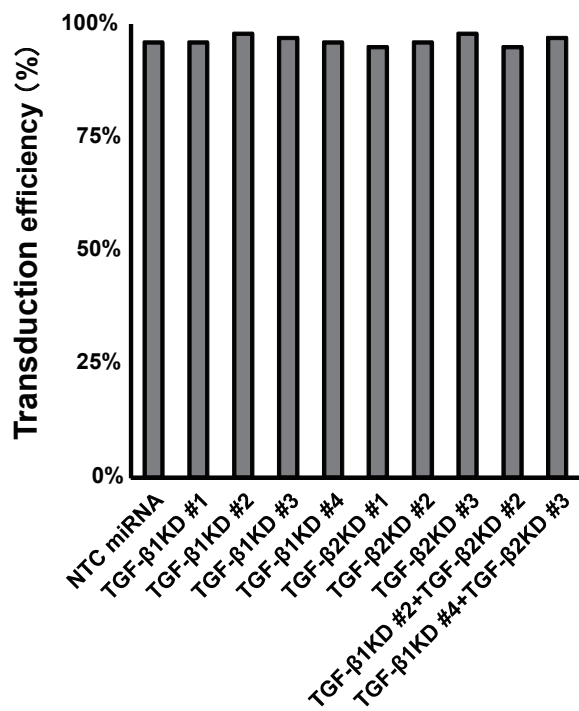

B HFL-1

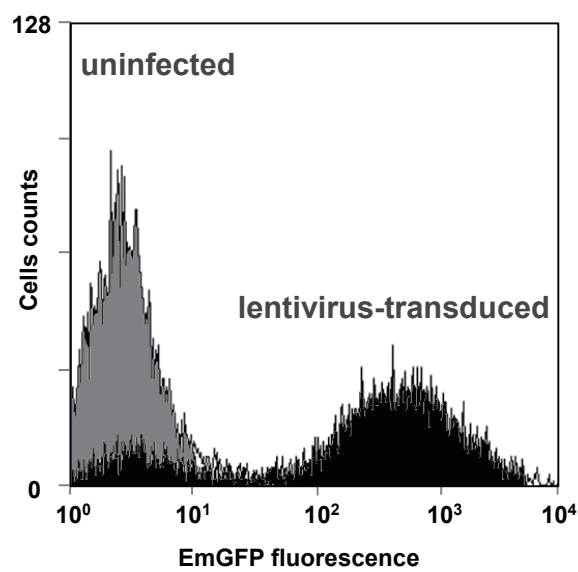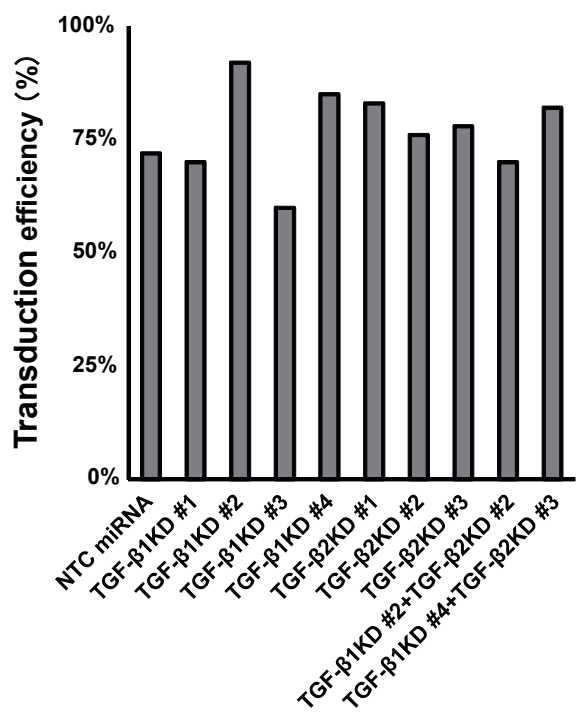

Supplement: Supplementary file 5 — Additional file 5: Figure S2: Transduction efficiency of lentiviral vectors. A: Transduction efficiency of miRNAs in A549 cells. Left: miRNA transduction was tracked by detecting EmGFP-positive cells using the FL-1 channel of a flow cytometer. A representative result of #2 miRNA transduction against TGF-β1 is shown. The grey and black peaks are from uninfected and lentivirus-transduced cells, respectively. Right: transduction efficiency of each miRNA. KD: knockdown. NTC: negative control. B: Transduction efficiency of miRNA in HFL-1 cells. (PDF 250 KB) [file 12885_2014_4753_MOESM5_ESM.pdf]

Supplementary Figure 3

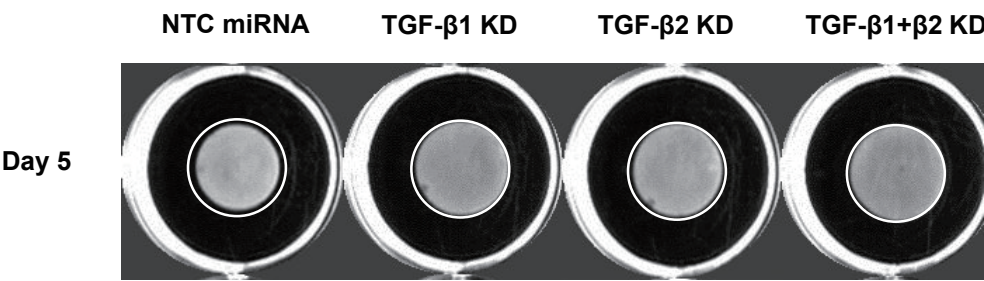

Supplement: Supplementary file 6 — Additional file 6: Figure S3: Collagen gel contraction assay. Photographs of the gels on day 5 in the experiments shown in Figure 5. Identically sized white circles in each well are shown to demonstrate the differences in gel size. (PDF 308 KB) [file 12885_2014_4753_MOESM6_ESM.pdf]
